# Structural and pharmacological insights into cordycepin for neoplasms and metabolic disorders

**DOI:** 10.3389/fphar.2024.1367820

**Published:** 2024-06-17

**Authors:** Jinming Zhang, Ziling Yang, Zhuo Zhao, Nan Zhang

**Affiliations:** ^1^ Department of Gastroenterology, First Hospital of Jilin University, Jilin University, Changchun, China; ^2^ Second Hospital of Jilin University, Jilin University, Changchun, China

**Keywords:** cordycepin, tumour, metabolic disorder, tumour microenvironment, hyperlipidaemia, diabetes

## Abstract

Cytotoxic adenosine analogues were among the earliest chemotherapeutic agents utilised in cancer treatment. Cordycepin, a natural derivative of adenosine discovered in the fungus Ophiocordyceps sinensis, directly inhibits tumours not only by impeding biosynthesis, inducing apoptosis or autophagy, regulating the cell cycle, and curtailing tumour invasion and metastasis but also modulates the immune response within the tumour microenvironment. Furthermore, extensive research highlights cordycepin’s significant therapeutic potential in alleviating hyperlipidaemia and regulating glucose metabolism. This review comprehensively analyses the structure-activity relationship of cordycepin and its analogues, outlines its pharmacokinetic properties, and strategies to enhance its bioavailability. Delving into the molecular biology, it explores the pharmacological mechanisms of cordycepin in tumour suppression and metabolic disorder treatment, thereby underscoring its immense potential in drug development within these domains and laying the groundwork for innovative treatment strategies.

## 1 Introduction

Cordyceps, a fungal organism, demonstrates an impressive ability to parasitize arthropods, particularly ghost moth larvae. This fungus consists of two main parts: the fruiting body, which produces spores, and the supporting mycelium. As autumn transitions to winter, the fungus insidiously invades and consumes the larvae from within. With the onset of summer, a spore-releasing stalk emerges from what was once the caterpillar’s head (Li et al., 2019).

This fascinating parasitic interaction is known in Chinese culture as “winter worm, summer grass.” Through evolutionary adaptation, Cordyceps has developed complex mechanisms to bypass and undermine the host’s immune defences, enabling its successful colonization within the unsuspecting host (Cunningham et al., 1950; Zhu et al., 1998; Zhang et al., 2021). Esteemed in Eastern medicinal practices for its array of pharmacological properties, Cordyceps is heralded as a prized and potent traditional remedy (Paterson, 2008). The fruiting body of Cordyceps contains a plethora of active substances, including cordycepin (COR), adenosine, and cordyceps polysaccharides, among which cordycepin is the most active ingredient (Wu WD. et al., 2014).

Cordycepin, the first natural derivative of a nucleoside, was initially isolated from the pupal Cordyceps by Cunningham et al. Also known as 3’-deoxyadenosine (Cunningham et al., 1950), its molecular structure is 9-(3-deoxy-β-D-ribofuranosyl) adenine (Tuli et al., 2013; Tan et al., 2020). Cordycepin demonstrates multifaceted intracellular activity, including the inhibition of RNA synthesis, modulation of inflammatory mediator pathways, induction of apoptosis, and regulation of lipid and glucose metabolism, among other effects (Rich et al., 1965; Wong et al., 2010; Ashraf et al., 2020). Consequently, it demonstrates a broad spectrum of therapeutic potential in the management of neoplasms, hyperlipidaemias, bacterial or viral infections, inflammation, and oxidative damage (Ashraf et al., 2020). Notably, cancer and metabolic disorders, exemplified by diabetes and hyperlipidaemia, constitute an escalating global health burden. Existing treatments, albeit somewhat effective, are frequently associated with significant side effects, substantial costs, and limited efficacy in certain instances. Thus, the pursuit of alternative and complementary therapeutic strategies is of paramount importance (Lee et al., 2012; Wu C. et al., 2014; Ma et al., 2015; Ashraf et al., 2020).

Emerging evidence suggests that hyperlipidaemia and diabetes mellitus are significant contributors to the heightened risk of immunological dysregulation (Mozaffarian et al., 2015; Ke et al., 2023; Mahemuti et al., 2023; Nie et al., 2023). The quantification of systemic inflammation can be achieved through an array of biochemical and haematological markers. Among these, the Systemic Immune-Inflammation Index (SII)—a composite biomarker derived from the product of platelet and neutrophil counts divided by the lymphocyte count—stands out as a robust and innovative measure of inflammatory status (Hu et al., 2014; Tong et al., 2017). The SII serves as an indicator of both localized and systemic inflammatory responses (Wang et al., 2021; Zhang et al., 2022), and has gained recognition in oncological research for its prognostic value, as well as in cardiology as a predictor of coronary artery disease (Yang et al., 2020; Mahemuti et al., 2023). Notably, hyperlipidaemia and diabetes have been consistently associated with elevated SII levels (Mahemuti et al., 2023; Nie et al., 2023), underscoring their role as auto-inflammatory conditions. These diseases are characterized by chronic inflammation triggered by persistent metabolic stress, with the resultant low-grade inflammatory milieu posited as a pivotal driver of disease progression. Persistent inflammatory processes have been implicated in the pathogenesis of a multitude of complications, encompassing ocular, cardiovascular, and renal dysfunctions. These complications are often precipitated by deleterious fibrotic responses (Han et al., 2020; Ikeno et al., 2020; Guo et al., 2022; Deka and Li, 2023).

The multifaceted physiological impacts of cordycepin are predominantly attributed to its unique molecular structure, and several hypotheses have been posited by the scientific community to elucidate its mechanism of action. The predominant hypothesis suggests that cordycepin undergoes a biochemical transformation into cordycepin triphosphate (COR-tp) via a phosphorylation pathway (Wang et al., 2016). Owing to the structural similarity between COR-tp and Adenosine Triphosphate (ATP), the former is frequently misrecognized as ATP, resulting in its incorporation into enzymatic processes where it acts as a substitute for ATP (Hueng et al., 2017). This molecular mimicry is believed to underlie the diverse biological activities of cordycepin. This leads to abnormal purine metabolism, with the activity of ATP-targeted protein kinases being inhibited or aberrantly activated (Wu WD. et al., 2014; Tuli et al., 2014). Furthermore, cordycepin also has the potential to act as a ligand (Cheutin et al., 2002) and an RNA elongation inhibitor (Sakaguchi et al., 2004), specifically inhibiting transcription efficiency (Hueng et al., 2017). As PolyA polymerase mistakenly identifies COR-tp as ATP, mRNA adenylation may also be disrupted by COR-tp (Levenson et al., 1979).

**FIGURE 1:**
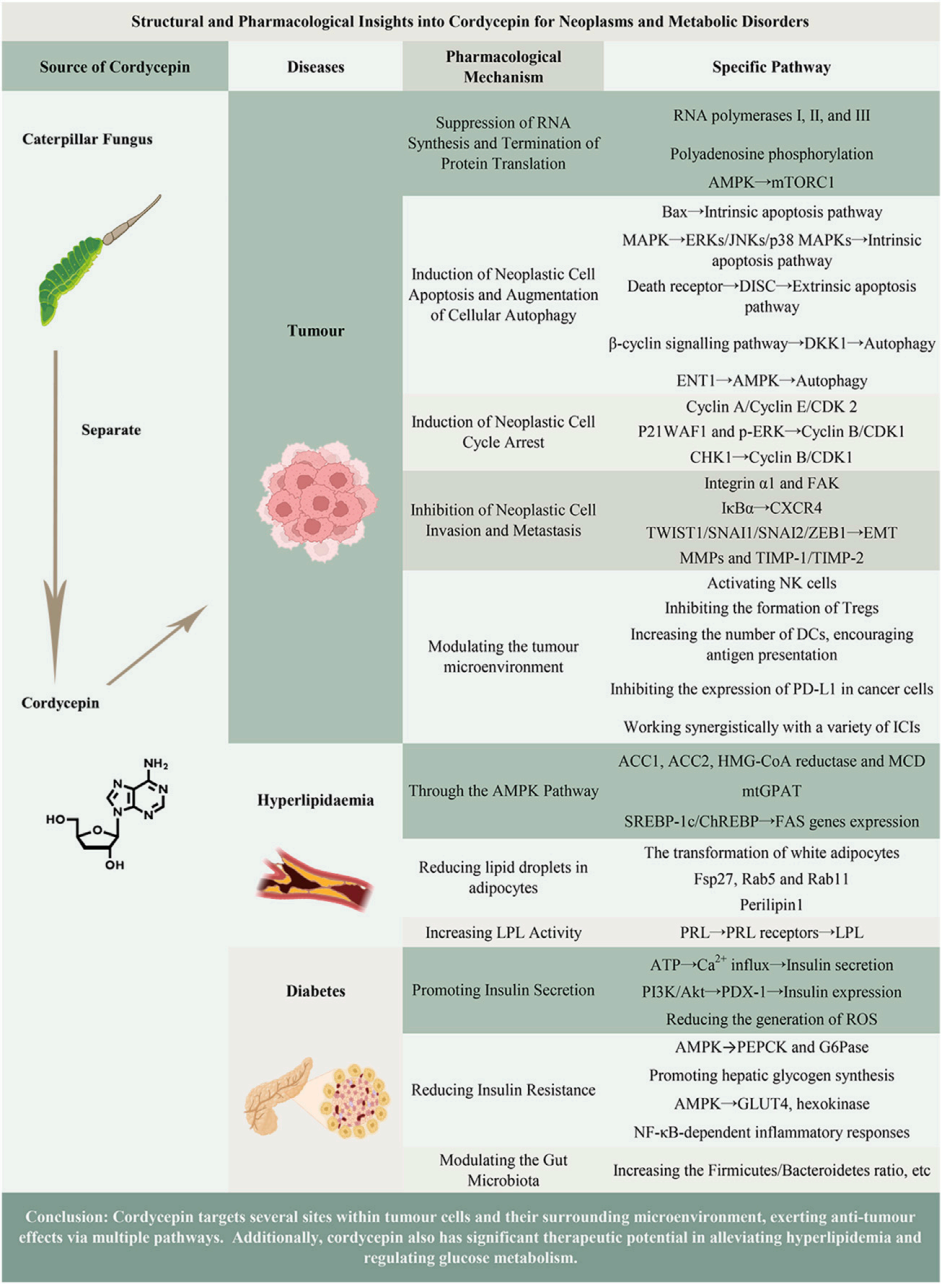
Graphical abstract of the main content of the article.

Given the complex biological functions and tremendous medicinal potential of cordycepin (Tuli et al., 2013; Wang et al., 2016), there is a notable gap in the literature. The current discussions on cordycepin’s pharmacokinetic characteristics, medicinal targets, and the functional roles of its analogues are insufficient. Consequently, this article aims to provide a concise summary of these crucial aspects. Furthermore, this manuscript will specifically concentrate on a meticulous and exhaustive review of the pharmacotherapeutic implications of cordycepin in the realms of oncology, hyperlipidaemia, and diabetes. This will facilitate the provision of novel insights within the framework of fundamental and evidence-based medicine, thereby paving the way for the evolution of innovative therapeutic strategies for patients afflicted with these conditions. The main content of this article is visually summarized in Figure 1.

## 2 Methods

We conducted a comprehensive literature search to identify articles exploring the relationship between cordycepin and tumours, hyperlipidaemia, and diabetes. We searched electronic databases including PubMed, Web of Science, SinoMed, and CNKI, covering the period from January 1950 to December 2023. This narrative review was guided by the Preferred Reporting Items for Systematic Reviews and Meta-Analyses (PRISMA) statement (Shamseer et al., 2015).

Following the guidelines of the 2020 PRISMA statement (Page et al., 2021), our research team assessed several key elements: research questions, eligibility criteria, information sources, search strategies, data collection, screening of selected scientific papers, and presentation of the main findings and conclusions, including the strengths and limitations of these studies.

Our inclusion criteria were as follows: articles written in English or Chinese, investigating the relationship between cordycepin or its analogs and tumours, hyperlipidaemia, and diabetes in basic research, clinical trials, and review articles, utilizing reliable research tools. Excluded articles comprised non-English or non-Chinese publications, non-peer-reviewed articles, and conference abstracts, the latter only used for snowball searching strategies.

Researchers performed an initial selection by screening titles and abstracts. Two researchers were responsible for each stage of study selection, removing duplicate entries, and categorizing studies as excluded or requiring further evaluation. All data were extracted by two researchers and cross-checked by another two. In cases of disagreement during study selection, we resolved differences through team discussions.

This methodology ensured a comprehensive and systematic screening of the literature to thoroughly investigate the relationship between cordycepin and tumours, hyperlipidaemia, and diabetes.

## 3 The structure-activity relationship of cordycepin and its analogues

**FIGURE 2:**
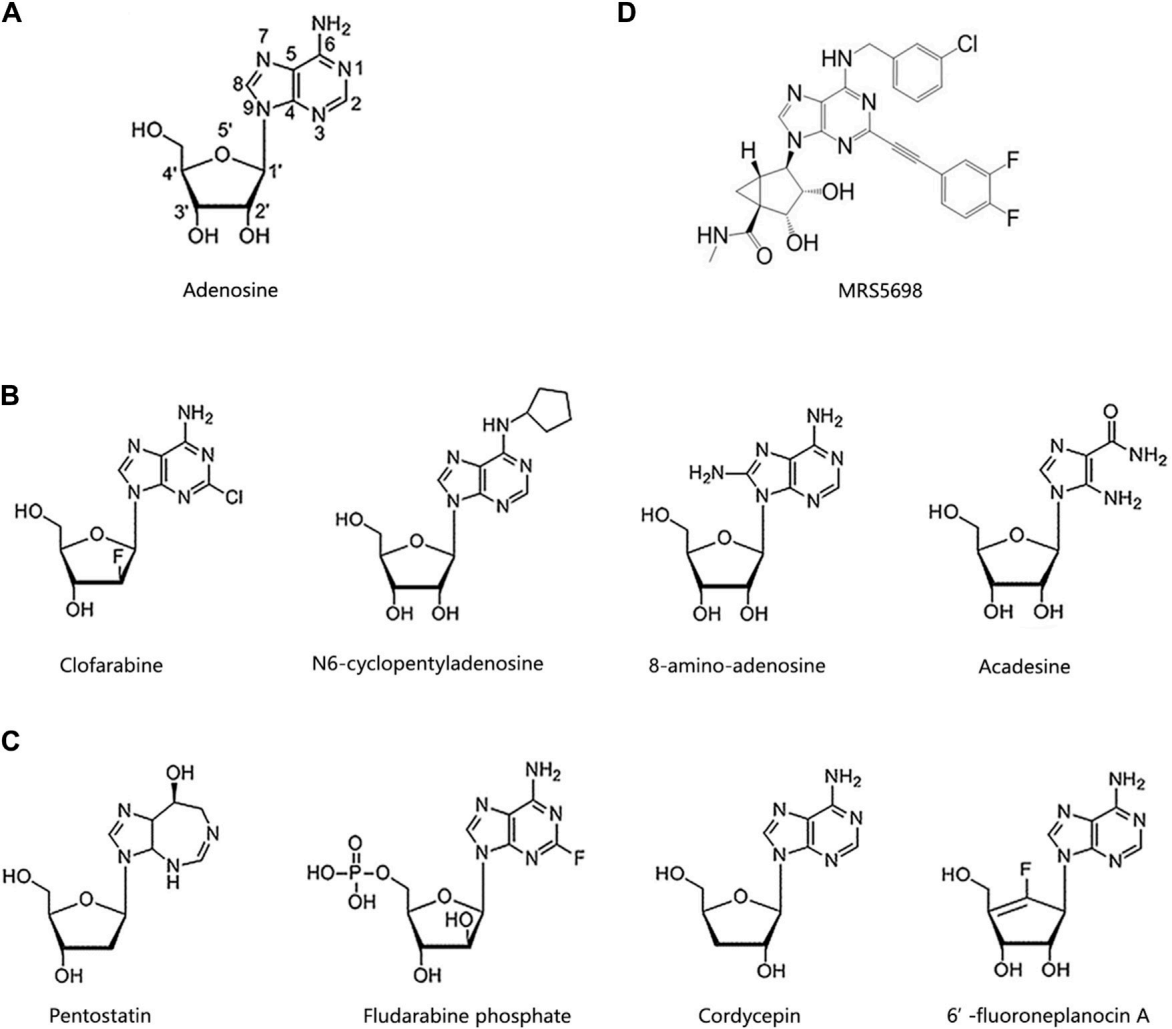
Chemical structures of cordycepin and its analogues. **(A)** The number of positions on the adenosine. **(B)** Adenosine derivatives primarily modified on the purine ring. **(C)** Adenosine derivatives primarily modified on the ribose ring. **(D)** MRS5698.

As previously mentioned, cordycepin was the first nucleoside natural derivative discovered, formed by replacing the hydroxyl group at the C3’ position of the adenosine ribose ring with a hydrogen atom. In fact, adenosine analogues like cordycepin have already been developed into marketed drugs or used as anticancer agents in numerous clinical trials. In this chapter, we will delve into the structure-activity relationship of these adenosine analogues to further elucidate their functional roles. In adenosine analogues, modifications to C2, N6, and C8 of adenosine, or C2’, C3’, C4’, and C5’ of ribose, confer varying anticancer activities (Figure 2A).

According to structure-activity relationship analysis, adenosine analogues with modifications on the adenine (purine) ring generally adhere to these rules (Figure 2B). Firstly, modifications at C2, particularly the introduction of halogen atoms at this position, demonstrate potent anticancer activities, with the length of the substituent being inversely related to anticancer effectiveness. Clofarabine, cladribine, and fludarabine are classic examples of such modifications (Galmarini et al., 2003; Lubecka-Pietruszewska et al., 2014; Song et al., 2016). Clofarabine, which acts a DNA polymerase inhibitor, has been approved for the treatment of multiple myeloma (MM), non-Hodgkin lymphoma, and various types of leukemia (Valdez et al., 2013; Hayes et al., 2017).

Secondly, the amino group at C6 is essential for maintaining anticancer activity. For instance, N6-cyclopentyladenosine, which alters the amino group at C6, shows reduced cytotoxicity compared to adenosine (Dastjerdi et al., 2016). Thirdly, modifications at C8, such as 8-amino-adenosine, have significant anticancer effects (Yang et al., 2018). 8-amino-adenosine, with an amino substitution at C8, reduces cellular ATP levels and inhibits mRNA synthesis, thereby inducing cytotoxic effects on tumour cells (Polotskaia et al., 2012).

Fourthly, alterations in the heteroaromatic ring of adenine, as opposed to the unmodified purine, enhance anticancer activity. For example, acadesine, an activator of AMP-activated protein kinase (AMPK), features a “break” in the heteroaromatic ring (Nie et al., 2017). It is approved for clinical treatment of cervical cancer, MM, mantle cell lymphoma, B-cell chronic lymphocytic leukemia, myelodysplastic syndrome, and acute myeloid leukemia. Additionally, it has demonstrated positive effects on malaria, myocardial infarction, diabetes, and during coronary artery bypass surgery (Nafz et al., 2007; Gonzalez-Girones et al., 2013; Cluzeau et al., 2020).

Modifications to the ribose ring of adenosine primarily occur at the C2’, C3’, C4’, and C5’ positions, as illustrated in Figure 2C. For example, pentostatin, which lacks a hydroxyl group at the C2’ position, is an effective inhibitor of both adenosine deaminase (ADA) and S-adenosylhomocysteine hydrolase (AHCY). It can inhibit DNA synthesis and induce adenosine receptor-dependent apoptosis (Dangvu et al., 1988; Sauter et al., 2008). Fludarabine phosphate, modified at the C4’ position, competes with dATP for incorporation into DNA to inhibit DNA synthesis (Sorscher et al., 2012). It is currently employed in clinical settings for treating neuroblastoma, metastatic melanoma, and various forms of leukemias. Additionally, this drug has shown effectiveness against conditions like arterial restenosis and congenital ichthyosis (Foran et al., 1999; Boogaerts et al., 2001; Tobinai et al., 2006).

A typical example of an adenosine analogue modified at the C3’ position is cordycepin, which has been extensively studied and undergone multiple clinical trials. The specific mechanisms by which it acts against cancer and metabolic disorders will be detailed later in the text. 6’-Fluoroneplanocin A, modified with a fluorine atom at the C5’ position, is the most effective inhibitor of AHCY and can inhibit the activity of vesicular stomatitis virus and tumour cells (Chandra et al., 2015).

In summary, it is clear that the adenosine derivative family, including cordycepin, constitutes effective anticancer agents. In-depth research into these compounds is bound to provide valuable options for novel anticancer drug development.

## 4 Pharmacokinetic

Understanding the pharmacokinetics of cordycepin is crucial for optimizing its clinical efficacy. The absorption, distribution, metabolism, and excretion (ADME) characteristics of cordycepin directly influence its bioavailability and therapeutic levels, impacting its effectiveness in clinical settings (Hawley et al., 2020). This section transitions into a detailed discussion on how these pharmacokinetic properties support its pharmacological actions in treating neoplasms and metabolic disorders. In fact, as demonstrated by its molecular structure, the metabolic pathways of cordycepin are quite similar to those of adenosine.

Cordycepin administration can be conducted orally or intravenously, though its oral absorption is notably limited and inconsistent due to rapid metabolism by adenosine deaminase (Guan et al., 2020). When cordycepin is given orally to rodents at a dosage of 100 mg/kg, it leads to a relatively low peak plasma concentration, averaging around 0.004 ± 0.001 μg/mL. On the other hand, intravenous administration significantly and rapidly increases plasma cordycepin levels, but this is followed by a quick decrease. For example, administering a 10 mg/kg dose intravenously in rats results in a peak concentration of 2.1 ± 0.9 μg/mL (Qi et al., 2023).

The pharmacokinetics of cordycepin are marked by its wide distribution volume, suggesting its ability to permeate various body tissues. Notably, cordycepin can cross the blood-brain barrier, thereby gaining access to the central nervous system. It also permeates several other vital structures, including the liver, kidneys, spleen, lungs, heart, and muscles (Wei et al., 2021). The distribution volume of cordycepin, determined following a 10 mg/kg intravenous dose in rat models, was recorded at 8.80 ± 4.30 L/kg (Qi et al., 2023).

Metabolically, cordycepin undergoes primary degradation via the enzymatic actions of ADA and adenosine kinase (Hawley et al., 2020). ADA catalyses the deamination of cordycepin to yield 3’-deoxyinosine, a metabolite devoid of activity, whereas adenosine kinase facilitates the phosphorylation of cordycepin, producing active metabolites that include the mono-, di-, and triphosphate derivatives (Yoon et al., 2018). The metabolite ratio depends on cordycepin concentration and enzymatic activity.

Cordycepin and its metabolites are primarily excreted in urine. The elimination half-life of cordycepin is extremely short and the clearance of cordycepin is exceptionally high, indicating rapid renal excretion. In rats, the elimination half-life and the clearance of cordycepin after intravenous administration at doses of 10 mg/kg were 5.1 ± 1.2 min and 1.16 ± 0.44 L/min/kg, respectively (Qi et al., 2023).

In order to augment the bioavailability and therapeutic efficacy of cordycepin, three primary strategies can be implemented: synergistic application of ADA inhibitors, structural alterations to generate cordycepin derivatives, and development of innovative drug delivery systems for its administration. ADA inhibitors, including pentostatin, erythro-9-(2-hydroxy-3-nonyl) adenine (EHNA), and natural compounds derived from certain traditional Chinese medicines, have the capacity to inhibit adenosine deamination reactions, regulate the concentrations of intracellular adenosine and deoxyadenosine, influence lymphocyte proliferation and functionality, and amplify the therapeutic impact of adenosine analogues (Davidson and Feigelson, 1956). Nevertheless, in animal studies, canines administered with a combination of cordycepin and pentostatin exhibited severe gastrointestinal and bone marrow toxicity, not observed when cordycepin was administered independently (Rodman et al., 1997).

Another approach to prevent the rapid degradation of cordycepin by ADA into 3’-deoxyinosine is to synthesize its derivatives through structural modification of cordycepin. The pentose ring’s hydroxyl group and the purine ring’s N-6 amino group are prevalent modification sites for cordycepin (Jin et al., 2022). For example, Pedro and colleagues employed *in vitro* molecular docking simulations—a computational chemistry method used to predict the interaction modes and binding strengths between two or more molecules, which is now widely applied in drug design and target identification processes (Grajales and Kar, 2023). They analysed the molecular affinity of 31 compounds with chemical structures similar to cordycepin towards A3 adenosine receptors (A3AR) agonists and ADA. They discovered that MRS5698 can bind to A3AR and inhibit cancer cell growth. However, its binding affinity to ADA is less than half of that of cordycepin, suggesting it would be less metabolized by ADA (Fong et al., 2019) (Figure 2D). Moreover, cordycepin oligomers, conforming to the general formula p5’(3’dA)-2’(p5’[3’dA])_n_ (*n* = 1–5), have been synthesized to bolster cordycepin’s stability and degradation resistance (Sawai et al., 1983).

Innovative drug delivery systems, including liposomes, nanoparticles, and microemulsions, are considered potential and efficacious strategies to augment cordycepin’s bioavailability and therapeutic efficacy (Dymek and Sikora, 2022; Lyu et al., 2022). Liposomes, composed of phospholipids and cholesterol, share a structural similarity with cell membranes (Dymek and Sikora, 2022; Lyu et al., 2022). As a drug delivery system, liposomes offer numerous advantages, including biocompatibility, biodegradability, the capacity to encapsulate both hydrophilic and hydrophobic drugs, low toxicity, protection of active agents, and the ability to deliver drugs both passively and actively without the need for surface modification (Urbinati et al., 2012; Qu et al., 2013). Wu et al. (Wu et al., 2016) prepared cordycepin-loaded liposomes using the ammonium sulfate gradient method. These liposomes significantly enhanced the inhibitory effect of cordycepin on the growth of H22 liver cancer tumours in mice at both low and high doses, administered via intravenous injection of 0.2 mL of 4, 2, 1 mg/kg cordycepin liposomes daily for 10 consecutive days, with no apparent adverse effects on the immune system.

Nanoparticles can protect encapsulated drug molecules from harsh conditions, such as chemical and enzymatic degradation, alter the pharmacokinetics and tissue distribution of drugs in the body, and enhance drug efficacy. Poly (lactic-co-glycolic acid) (PLGA) nanoparticles containing cordycepin, prepared via the double emulsion solvent evaporation method, ranged from 179 to 246 nm in size and sustained drug release for over 10 days at 37°C in pH 7.4 PBS, enhancing cellular uptake of cordycepin (at a concentration of 50 μg/mL for 4 h). These nanoparticles significantly increased cytotoxicity against human breast cancer MCF-7 cells, with an IC50 value of 16.79 μg/mL, compared to the free drug’s IC50 value of 47.84 μg/mL (Marslin et al., 2020). Furthermore, Kengkittipat and colleagues designed and produced bile acid core/shell chitosan shell hybrid nano-carriers centred on cordycepin using the solvent (ethanol) injection method. By incorporating bile salts into the nano-carriers, they protected them from gastrointestinal degradation, significantly improving the oral bioavailability of cordycepin (Kengkittipat et al., 2021).

However, it is worth noting that most studies on novel drug delivery systems are still at the experimental stage. Some inherent shortcomings of these carriers, such as imprecise control release and poor localization of liposomes, along with the need for improvement in drug loading and encapsulation efficiency of nanoparticles, should be considered. Therefore, scholars should focus more on new technologies, excipients, and formulations to promote further research and potentially clinical applications of cordycepin.

## 5 Anti-neoplastic efficacy

The escalating incidence and mortality rates of tumours have positioned them as a significant public health concern and a primary cause of death. In 2022, the United States alone reported approximately 1,918,030 new cancer cases and an estimated 609,360 cancer-related deaths (Siegel et al., 2022). This surge in tumour incidence and mortality rates over recent years underscores the urgent need for effective therapeutic strategies (Liu et al., 2003).

Tumours are characterised by uncontrolled and aggressive cellular proliferation, primarily driven by the downregulation of tumour suppressor genes and/or the upregulation of tumour promoter genes (Liu et al., 2003). This aberrant cellular behaviour underscores the complexity of tumour biology and the challenges associated with its management.

In contrast, cordycepin, a bioactive compound, has demonstrated significant anti-tumour properties. It has been reported to inhibit the growth of various cancer cell types, including those of lung, colon, and prostate cancers. The anti-tumour effects of cordycepin are mediated through multiple pathways, including inhibiting tumour growth, tumour cell proliferation, and metastasis, as well as regulating the tumour microenvironment (Nakamura et al., 2006; Lee et al., 2010; Lee et al., 2012). This multi-faceted approach to tumour inhibition highlights the potential of cordycepin as a promising candidate for cancer therapy.

### 5.1 Suppression of RNA synthesis and termination of protein translation

**FIGURE 3:**
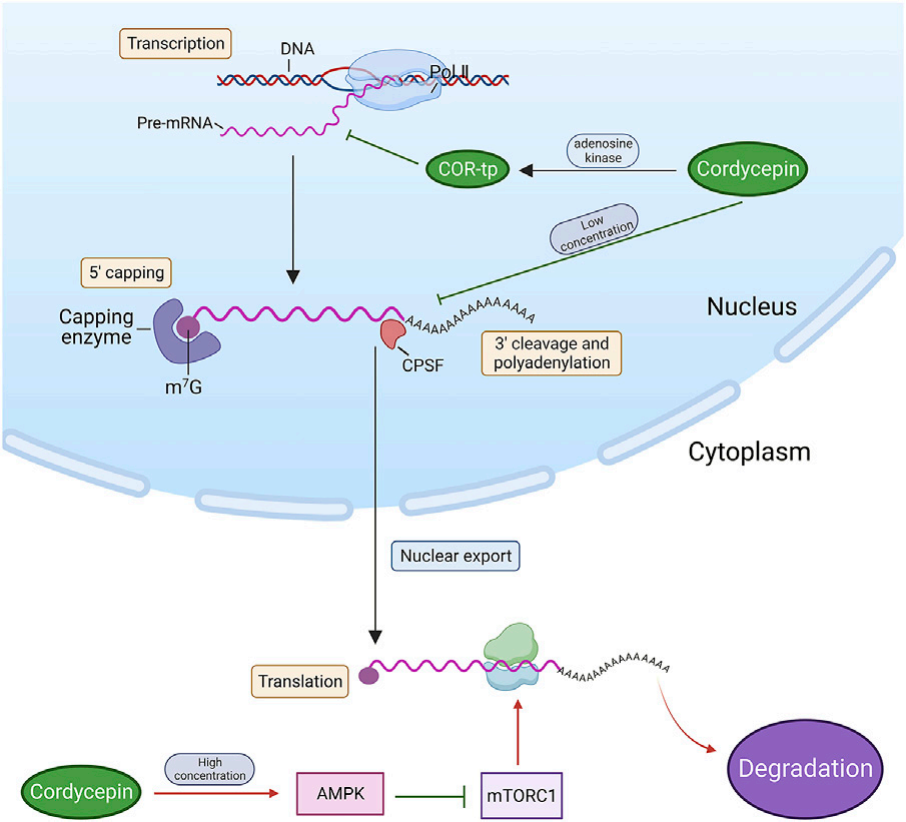
Cordycepin inhibits tumour cell growth by obstructing RNA synthesis and terminating protein translation. (Note: Red arrows denote an upstream factor inducing a downstream target, while green arrows signify an upstream factor inhibiting a downstream target. This arrow annotation format is maintained consistently throughout all figures in this review. Pol II: RNA polymerases II. m7G: N7-methylguanosine. CPSF: Cleavage and Polyadenylation Specificity Factor).

As previously discussed, adenosine kinase facilitates the phosphorylation of cordycepin into a phosphate derivative, structurally similar to Adenosine Monophosphate (AMP), Adenosine Diphosphate (ADP), and ATP. This derivative, COR-tp, can be mistakenly identified as ATP, thereby becoming involved in RNA synthesis and inhibiting the functionality of intracellular RNA polymerases I, II, and III, culminating in the cessation of RNA chain synthesis (Klenow, 1963; Hawley et al., 2020). In mammalian fibroblasts, low cordycepin concentrations impede polyadenosine phosphorylation, truncate the poly(A) tails of certain mRNAs, and obstruct the processing of mRNA at the 3’ end, thus inhibiting RNA synthesis (Wong et al., 2010). Conversely, elevated cordycepin concentrations can stimulate the 5’-adenosine monophosphate-activated protein kinase—a highly conserved serine/threonine kinase and a pivotal regulator of intracellular energy metabolic homeostasis (Wong et al., 2010; Hawley et al., 2020)—in the form of cordycepin monophosphate (COR-mp). Through the activation of the intracellular AMPK signalling pathway, cordycepin suppresses the mammalian target-of-rapamycin complex-1 (mTORC1) pathway, thereby inhibiting protein translation. This ultimately results in the suppression of lung cancer cell survival, migration, and invasion (Wei et al., 2019). (Figure 3)

### 5.2 Induction of neoplastic cell apoptosis and augmentation of cellular autophagy

Apoptosis, a programmed cellular death mechanism, is typically instigated by both endogenous or exogenous stimuli. Endogenous stimuli predominantly encompass reactive oxygen species and endoplasmic reticulum stress, which can modify the permeability of the outer mitochondrial membrane by activating pro-apoptotic proteins within the B-cell Lymphoma (BCL)-2 protein family. This, in turn, governs the release of pro-apoptotic factors from the mitochondria, such as cytochrome C (Cyt C), apoptosis-inducing factor (AIF), and Smac/DIABLO, thus activating the downstream caspase cascade reaction (Elmore, 2007). Conversely, exogenous stimuli primarily refer to external cellular factors, including cytokines, growth factors, hormones, viruses, bacteria, and others. These factors, by binding to the death receptor (DR) on the cellular surface and recruiting junction proteins to form the death-inducing signalling complex (DISC), activate the downstream apoptotic pathway (McIlwain et al., 2015). Extensive research has elucidated that cordycepin initiates the intrinsic apoptotic pathway within the body. This process commences with the translocation of the Bax protein, which subsequently facilitates the release of cytochrome c from the mitochondria into the cytoplasm, thus precipitating the activation of caspase-9 (Choi et al., 2011; Liao et al., 2015). The binding affinity of cordycepin with BCL-2 has been confirmed through simulated molecular docking experiments. The binding site is located within the binding pocket of the protein structure (Tania et al., 2019). Additionally, it is important to highlight that cordycepin may have a role in enhancing death receptor-mediated extrinsic apoptotic pathways. This nucleoside analogue is capable of inducing caspase-3 activation by directly cleaving active caspase-8, a component of the DR signalling pathway. This leads to a series of caspase reactions, essential for the progression of apoptosis (Lee et al., 2013; Lee et al., 2014).

The Mitogen-Activated Protein Kinase (MAPK) pathway, encompassing crucial proteins such as Extracellular Signal-Regulated Kinases (ERKs), c-Jun N-terminal kinases (JNKs), and p38 MAPK, plays a vital role in various cellular functions (Lee et al., 2009). Research by Baik et al. demonstrated that inhibiting ERK, JNK, and p38 MAPK reduced the effectiveness of cordycepin in inhibiting the growth of human glioblastoma U87MG cells (Baik et al., 2016). This highlights the significance of the MAPK pathway in the antiproliferative effects of cordycepin. Moreover, studies by Pan et al. showed that decreasing p38 MAPK activity reduced apoptosis in mouse testicular mesenchymal tumour cells treated with cordycepin, indicating the p38 MAPK pathway’s crucial involvement in cordycepin-induced apoptosis (Pan et al., 2015).

**FIGURE 4:**
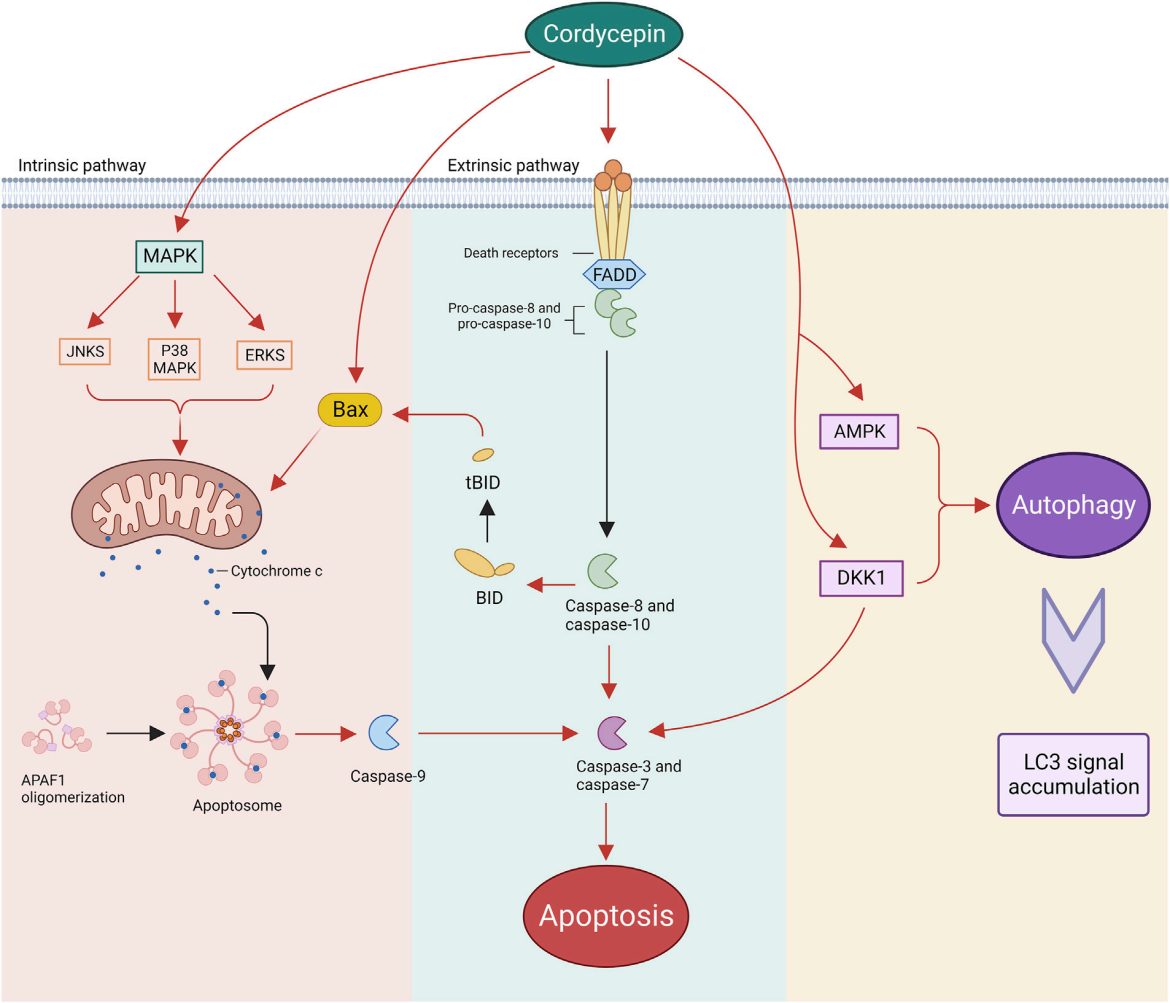
Cordycepin instigates neoplastic cell apoptosis through both intrinsic and extrinsic pathways, while also enhancing autophagy within these malignant cells. (Note: APAF1: Apoptotic Protease Activating Factor 1. Bax: BCL-2-associated X Protein. BID: BCL-2 Homology-3 Interacting Domain Death Agonist. tBID: truncated BID. FADD: Fas-associated Protein with Death Domain).

Autophagy, a cellular mechanism prevalent from yeast to mammals, is primarily tasked with degrading and recycling cellular components, and occasionally entire cells, via the lysosomal pathway (Amenta and Brocher, 1981). In tumour cells, autophagy plays a dual role, acting both as a promoter and an inhibitor of tumourigenesis. Specifically, autophagy suppresses tumour growth during the initial stages of tumourigenesis, but paradoxically, it facilitates tumour progression in the later stages and during treatment (Zhang and Ney, 2009; Chen et al., 2021). Consequently, autophagy presents a viable intervention strategy for tumour prevention and treatment across all stages. Cordycepin, a bioactive compound, may play a pivotal role in tumour therapy through its influence on the autophagy mechanism. In ovarian cancer cells, cordycepin instigates Dickkopf-related protein 1 (DKK1)-associated autophagy by inhibiting the β-cyclin signalling pathway. This autophagy process subsequently promotes the cleavage of caspase-3, leading to apoptosis (Jang et al., 2019). Another study revealed that cordycepin penetrates the ovarian cancer cell membrane via equilibrative nucleoside transporters (ENT1) and activates AMPK, which signals downstream target proteins to induce autophagy and trigger cell death (Yoon et al., 2022). Furthermore, a study indicated that the signals of microtubule-associated protein light chain 3 (LC3) were significantly amplified in human gestational choriocarcinoma JAR cells following cordycepin treatment. The accumulation of LC3 signals is often a marker of accelerated autophagy flux. In a subsequent study, JAR cells were co-treated with cordycepin and the autophagy inhibitor chloroquine, resulting in increased cell survival. This outcome corroborates that cordycepin induces autophagy activation and inhibits cell growth in JAR cells (Wang et al., 2020). Currently, while some mechanisms have been elucidated in detail, further *in vitro* and *in vivo* studies are needed to fully elucidate the role of cordycepin-activated autophagy in tumour therapy (Figure 4).

### 5.3 Induction of neoplastic cell cycle arrest

The regulation of the cell cycle is orchestrated by an intricate network of cyclins, cyclin-dependent kinases (CDKs), and cyclin-dependent kinase inhibitors (CDKIs). Among these, CDK1, 2, 4, and 6 are pivotal in facilitating cell cycle progression, a process that is notably disrupted in cancerous cells. Presently, the therapeutic targeting of the cell cycle is perceived as a promising avenue for tumour treatment.

Cordycepin exhibits varying degrees of cell cycle arrest effects across different tumour cell types (Lin et al., 2018; Tania et al., 2019; Wang et al., 2019; Chang et al., 2020; Li et al., 2020; Zheng et al., 2020; Lee et al., 2021; Lee et al., 2022; Shi et al., 2022). In certain studies, cordycepin has been observed to induce S phase arrest in tumour cells. For example, cordycepin has been shown to suppress CDK2 expression and concurrently reduce cyclin E and cyclin A2 levels in leukaemia, pancreatic cancer, and cholangiocarcinoma cells. Tania and colleagues’ molecular docking experiments also confirmed the binding of cordycepin with CDK-2, Cyclin-E1, and Cyclin-A2, the binding affinity to CDK-2 being the highest (Tania et al., 2019). The drug’s position within the binding pocket was further verified through superposition analysis. This leads to the arrest of tumour cells in the S phase, given the pivotal role of CDK2 in the transition from G1 to S phase and in S phase progression (Wang et al., 2014; Liao et al., 2015; Li et al., 2020).

**FIGURE 5:**
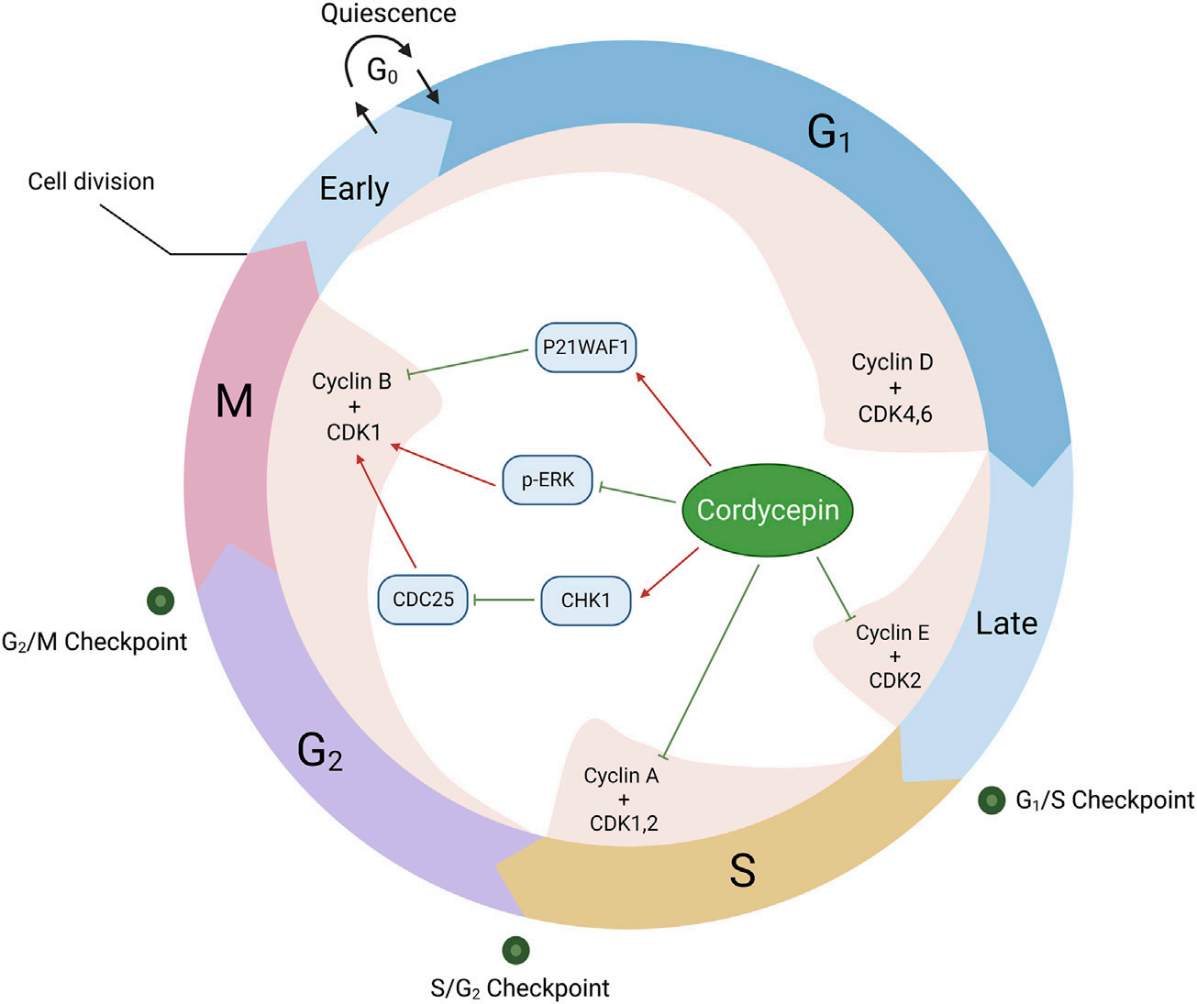
Cordycepin provokes cell cycle inhibition in neoplastic cells. (Note: CDC25: Cell Division Cyclin 25)

In addition, studies have noted that cordycepin induces a G2/M phase arrest in tumour cells, potentially through the suppression of cyclin B/CDK1 expression via multiple mechanisms. These encompass the enhancement of P21WAF1 gene expression in both human bladder and colon cancer cells, and the downregulation of p-ERK expression in oesophageal cancer cells (Lee et al., 2009; Lee et al., 2010; Xu JC. et al., 2019). Additionally, in HeLa cells, cordycepin has been shown to promote a G2/M phase arrest through the activation of the checkpoint kinase 1 (CHK1)/cyclin B-CDK1 complex pathway (Seong da et al., 2016). This evidence underscores the potential of cordycepin as a potent anti-cancer agent (Figure 5).

### 5.4 Inhibition of neoplastic cell invasion and metastasis

Tumour metastasis, the process by which tumour cells disseminate from the primary site and progressively colonise distant organs, is the primary cause of mortality in over 90% of tumour patients (Robinson et al., 2017).

Cordycepin has been demonstrated to significantly impede the migration of human glioblastoma cell lines U87MG and LN229 in invasion and wound-healing assays, as reported in a study (Hueng et al., 2017). This study also revealed a reduction in the expression of integrin α1, focal adhesion kinase (FAK), and phosphorylated FAK, suggesting that cordycepin may inhibit the metastatic spread of human glioblastoma cells by influencing lysosomal degradation and protein phosphatase activation.

Previous research has indicated that the CXC chemokine receptor type 4 (CXCR4) is highly expressed in hepatocellular carcinoma tissues, and that these cells preferentially metastasise to organs and tissues with high levels of CXCR4 ligand stromal cell-derived factor 1 (SDF1) expression (Wang et al., 2018). Guo et al.(Jang et al., 2019) demonstrated that cordycepin inhibited the activation and nuclear translocation of p65 by down-regulating the phosphorylation of NF-κB inhibitor α (IκBα), which subsequently downregulated CXCR4 and inhibited the migration and invasion of hepatocellular carcinoma cells *in vitro*.

The epithelial-to-mesenchymal transition (EMT) is considered to play a pivotal role in tumour metastasis. A cohort of pivotal transcription factors, encompassing Twist Family Basic Helix-loop-helix Transcription Factor 1 (TWIST1), Snail Family Transcriptional Repressor 1 (SNAI1), Snail Family Transcriptional Repressor 2 (SNAI2), and Zinc Finger E-Box Binding Homeobox 1 (ZEB1), is instrumental in orchestrating the epithelial-mesenchymal transition (EMT). Empirical evidence indicates that cordycepin markedly attenuates the transcriptional expression of these factors within triple-negative breast cancer cells. This reduction posits that cordycepin may impede cellular migration and invasion through modulation of these transcription factors’ activities (Kang and Massagué, 2004; Asaduzzaman Khan et al., 2017; Wei et al., 2022).

Matrix metalloproteinases (MMPs), proteolytic enzymes that degrade components of the extracellular matrix, are implicated in the degradation of this matrix and are associated with oncogenic proliferation, dissemination, and invasiveness. The inhibition of MMPs is postulated to be a therapeutic strategy in mitigating tumour progression and expansion (Jiang and Li, 2021). Observations have documented that cordycepin diminishes MMP-9 activity and restricts the invasive potential of tumour cells in instances of human lung cancer and oral squamous carcinoma (Jiang and Li, 2021; Binlateh et al., 2022). Additionally, research conducted by Jeong et al. elucidated that cordycepin mitigated the invasiveness and metastatic potential of human prostate cancer cells LNCaP by reducing the activities of tight junction proteins and MMPs. Treatment with cordycepin also resulted in an upregulation of tissue inhibitors of matrix metalloproteinases 1 (TIMP-1) and TIMP-2, which are integral to the modulation of tumour metastasis (Jeong et al., 2012). These findings highlight the potential therapeutic value of cordycepin in cancer treatment.

### 5.5 Modulation of the tumour microenvironment

In addition to its role as a direct inhibitor of tumours, as previously described, the impact of cordycepin treatment on the tumour microenvironment (TME) is increasingly gaining attention within the academic community, yet, this aspect has not been systematically summarized. The TME refers to the environment surrounding tumour cells, including nearby blood vessels, immune cells, extracellular matrix, fibroblasts, and other various cell types and molecules. This environment plays a critical role in tumour growth, spread, and response to treatment (Chen et al., 2023). Cordycepin has been found to modulate the TME through various pathways of immune regulation.

Cordycepin can directly regulate the infiltration of immune cells in the TME. For example, after treatment with cordycepin, natural killer (NK) cells demonstrated enhanced proficiency against cholangiocarcinoma cells *in vitro*, leading to significantly increased cancer cell death (Panwong et al., 2021). In a breast cancer mouse model, cordycepin has been shown to inhibit the differentiation of T cells into regulatory T cells (Tregs) in the spleen, thereby delaying tumour growth (Jeong et al., 2013). In a colon cancer mouse model, cordycepin treatment not only led to an increase in the number of dendritic cells (DCs) within the TME but also encouraged DCs to present antigens to CD8^+^ T cells and CD4^+^ T cells, enhancing the anti-tumour response. Furthermore, the research team discovered that cordycepin treatment could weaken the interactions between DCs and Tregs, which may help inhibit the immunosuppressive pathways in the TME (Chen et al., 2024).

Cordycepin can also alter the immunosuppressive environment within the TME. On one hand, cordycepin directly affects the expression of immune checkpoint molecules. For instance, Shaoxian Wu and others, through both *in vivo* and *in vitro* experiments, found and confirmed that cordycepin significantly reduces the expression of PD-L1 in mouse colorectal cancer cells, inhibiting tumour immune escape (Wu et al., 2023). More notably, cordycepin has been found to synergistically interact with various immune checkpoint inhibitors (ICIs), complementing their advantages to enhance cancer immunotherapy.

For example, previous studies have demonstrated that in sepsis, Alzheimer’s disease, and the colon cancer TME, cordycepin weakens the function of M1 macrophages (pro-inflammatory/anti-tumour) and promotes the polarization of macrophages towards the M2 phenotype (anti-inflammatory/pro-tumour) (Wei et al., 2021; Chen et al., 2022). Based on this, Feng Chen and colleagues developed a combined treatment strategy of cordycepin with anti-CD47 antibodies. Using single-cell RNA sequencing (scRNA-seq) and flow cytometry, they discovered and confirmed that the combined treatment of cordycepin and anti-CD47 antibodies can reactivate macrophages and reverse macrophage polarization, significantly enhancing cordycepin’s tumour suppression effect and extending the progression-free survival of patients with gastrointestinal malignancies (Feng et al., 2023). Similarly, Lujun Chen and others used the MC38 and CT26 tumour models to evaluate the therapeutic effects of cordycepin, CTLA-4 blockade, and their combination therapy. The results showed that the combination of cordycepin with CTLA-4 blockade significantly improved the efficacy and exhaustion state of infiltrating CD8^+^ T cells in the TME, reduced the number of Foxp3+ Tregs, and enhanced the anti-tumour immunity mediated by CD8^+^ T cells in the TME (Chen et al., 2023).

TIGIT is an immune checkpoint within the immunoglobulin superfamily that has recently gained significant attention and is widely expressed on CD8^+^ tumour-infiltrating lymphocytes (TILs), NK cells, helper T (Th) cells, and Tregs across various cancers, including melanoma, non-small cell lung cancer, colorectal cancer, hepatocellular carcinoma, and gastric cancer (Johnston et al., 2014; Chauvin et al., 2015; Inozume et al., 2016; Thommen et al., 2018; Hu et al., 2020; Xu et al., 2020; Ostroumov et al., 2021). Due to the relatively minimal immune-related adverse effects found with TIGIT blockade (Levin et al., 2011), the combination inhibition of TIGIT and PD-1/PD-L1 represents a highly promising dual immune checkpoint inhibitor therapy. Rongzhang Chen and colleagues discovered that cordycepin plays a critical role in the effective anti-tumour action of anti-TIGIT therapy by upregulating the expression of Cd226 on TILs. Additionally, they observed that the combined treatment of cordycepin and anti-TIGIT could enhance the activation of NK cells and the activity of CD8^+^ TILs, reduce the expression of exhaustion marker genes in NK cells and CD8^+^ TILs, further supporting the anti-tumour potential of cordycepin in combination with TIGIT blockade (Chen et al., 2024).

## 6 Anti-hyperlipidaemic efficacy

Hyperlipidaemia, marked by irregular lipid levels in the blood, encompasses increased total cholesterol (TC), triglycerides (TG), and low-density lipoprotein cholesterol (LDL-c), along with decreased high-density lipoprotein cholesterol (HDL-c). It is a crucial risk determinant for cardiovascular pathologies like atherosclerosis and coronary heart disease (Dembowski et al., 2022).

Numerous studies (Guo et al., 2010; Gao et al., 2011; Wu C. et al., 2014) have confirmed the therapeutic efficacy of cordycepin in alleviating hyperlipidaemia. Yet, the exact mechanism is still under discussion. The widely accepted theory posits that cordycepin mainly regulates lipid metabolism through the AMPK pathway (Das and Chakrabarti, 2005; Ashraf et al., 2020). As mentioned earlier, COR-mp can act as an AMP analogue, activating AMPK and initiating subsequent cascade reactions. Wang and colleagues generated a potential molecular docking model of AMPK and COR-mp using AutoDock 3.05. They discovered that COR-mp is located at the centre of the binding pocket, with hydrophobic interactions and hydrogen bonds serving as the stabilizing forces for this protein-ligand interaction (Wang et al., 2010).

AMPK inhibits the synthesis of fatty acids and cholesterol by phosphorylating acetyl-CoA carboxylase 1 (ACC1) and 3-hydroxy-3-methylglutaryl-CoA (HMG-CoA) reductase, thereby rendering them inactive (Viollet et al., 2009). Additionally, AMPK-induced phosphorylation of ACC2 results in an upsurge in fatty acid oxidation. ACC1 is the rate-limiting enzyme for malonyl-CoA synthesis, a crucial substrate for fatty acid biosynthesis and a potent inhibitor of fatty acid oxidation. AMPK also augments malonyl-CoA decarboxylase (MCD) activity, further diminishing hepatic malonyl-CoA levels (Assifi et al., 2005).

**FIGURE 6:**
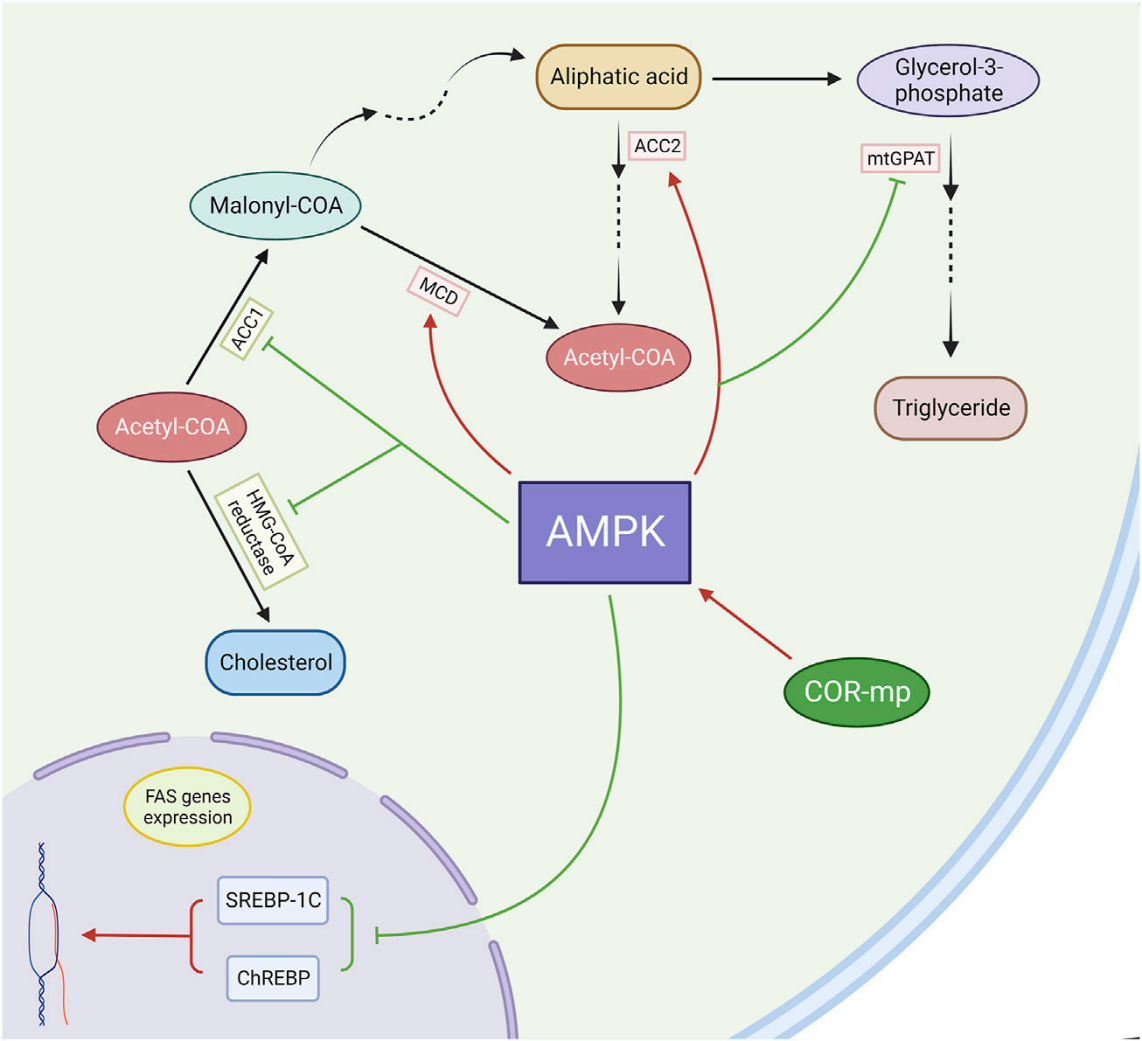
Cordycepin modulates lipid metabolism through the mediation of the AMPK pathway. (Note: FAS: Fatty Acid Synthase).

Hypertriglyceridemia has been observed in both systemic and liver-specific AMPKα2−/− mice, and the infusion of the AMPK activator AICAR (5-aminoimidazole-4-carboxamide-1-β-D-ribofuranoside) has been shown to reduce plasma TG levels (Bergeron et al., 2001; Andreelli et al., 2006). Furthermore, AMPK suppresses the expression of fatty acid synthase (FAS) genes by attenuating the actions of the transcription factors SREBP-1c and ChREBP (Kawaguchi et al., 2002; Foretz et al., 2005; Hu et al., 2021). Additionally, AMPK inactivates mitochondrial glycerol-3-phosphate acyltransferase (mtGPAT), thereby regulating acyl-CoA channelling towards β-oxidation and away from glycerolipid biosynthesis (Muoio et al., 1999). (Figure 6)

Beyond the AMPK pathway, several researchers have suggested alternative mechanisms through which cordycepin may influence lipid metabolism. Xu et al. demonstrated that cordycepin could facilitate the transformation of white adipocytes into beige and brown adipocytes, thereby enhancing the thermogenic capacity of these cells in a dose-dependent manner, which in turn promotes lipid catabolism (Xu H. et al., 2019). Concurrently, their team identified a downregulation in the expression of lipid droplet-associated genes such as Fat-Specific Protein (FSP)-27, Rab-5, Rab11, and Perilipin1 in adipose tissues. They postulated that cordycepin may impede lipid droplet formation via the downregulation of genes like FSP27, Rab5, and Rab11, and stimulate lipolysis by suppressing Perilipin1 (Xu H. et al., 2019).

In a separate study, Li et al. substantiated the inhibitory effect of cordycepin on prolactin (PRL) secretion by assessing PRL secretion in GH3 cells. They hypothesized that this pathway could also contribute to the lipid-lowering properties of cordycepin (Li et al., 2018). Prolactin, a hormone secreted by the anterior pituitary gland, plays a pivotal role in mammary gland development and lactation (Bole-Feysot et al., 1998). Elevated blood prolactin concentrations have been shown to decrease lipoprotein lipase (LPL) activity in adipose tissue via functional PRL receptors (Hamosh et al., 1970; Zinder et al., 1974; Jensen et al., 1994). LPL is an enzyme that binds to acetylheparan sulfate proteoglycans in the capillary wall, facilitating the hydrolysis of triglycerides in chylomicrons and very low-density lipoproteins, thereby aiding in the removal of triglycerides from the circulation and supplying free fatty acids to various tissues (Zechner et al., 2000).

The conventional therapies for hyperlipidaemia primarily include statins, fibrates, and niacin, which effectively reduce LDL-c and triglycerides and increase HDL-c. However, these treatments often come with side effects such as liver toxicity, muscle problems, and increased blood sugar levels (Pollex et al., 2008). In contrast, cordycepin offers a potentially safer profile with fewer adverse effects, as evidenced by its long-standing use in traditional medicine and recent clinical evaluations.

For example, Sun et al. found through nuclear magnetic resonance-based metabolomics research that cordycepin has a more beneficial lipid-lowering effect on the liver compared to simvastatin. While simvastatin primarily lowers blood lipids, it causes almost no change in the low-molecular-weight metabolites that are crucial for glucose/glycogen and lipid metabolism in the liver. However, in the livers of hyperlipidaemic hamsters treated with cordycepin, not only a similar lipid-lowering effect can be observed, but also a decrease in the signals of lactate, alanine, acetate, and glutamine, and an increase in the signals of choline compounds and glycogen. This indicates that after the administration of cordycepin, protein degradation in muscles and glucose production in the liver were inhibited in hyperlipidaemic hamsters, with energy expenditure shifting towards lipid oxidation, demonstrating cordycepin’s protective effect on the liver under conditions of fatty liver. (Sun et al., 2011).

Furthermore, as mentioned above, cordycepin’s mechanism, primarily through the AMPK pathway activation, differentiates it from statins which inhibit HMG-CoA reductase. This difference suggests potential synergies when used in combination, possibly enhancing lipid regulation while minimizing the risk of side effects associated with traditional therapies. Continuing research into these synergistic effects could provide a basis for integrating cordycepin into standard hyperlipidaemia management protocols, offering a comprehensive approach that leverages the strengths of both traditional and modern therapies.

## 7 Hypoglycemic efficacy

Diabetes mellitus is a multifaceted clinical syndrome typified by metabolic aberrations within the organism, predominantly characterised by chronic hyperglycaemia, a consequence of either absolute or relative insulin deficiency (Ding et al., 2018). The etiology of this endocrine metabolic disorder is intricate, implicating a multitude of factors, and poses a significant threat to human health (Antu et al., 2014). Presently, the dysfunction of pancreatic β-cells, insensitivity of insulin receptors, and insulin resistance are universally acknowledged as the principal pathogenic factors contributing to the onset of diabetes (Stuhlmann et al., 2018).

Cordycepin has been substantiated as an efficacious agent in the regulation of glucose metabolism and the mitigation of diabetic symptoms, as evidenced by a multitude of studies (Shin et al., 2009; Zhang et al., 2009; Ma et al., 2015; Sun et al., 2022; Liu et al., 2023; Yan et al., 2023). Ma et al. demonstrated that cordycepin treatment in alloxan-induced diabetic mice significantly decreased plasma glucose levels, mitigated common diabetic symptoms such as hyperphagia, irritability, thirst, and substantial weight loss, and enhanced performance in the oral glucose tolerance test (OGTT) (Ma et al., 2015). OGTT is a critical diagnostic tool for diabetes, assessing the functionality of pancreatic β-cells and the body’s capacity to regulate blood glucose (Matsuda and DeFronzo, 1999).

From a molecular biology standpoint, cordycepin’s regulation of diabetes can be attributed to several mechanisms. Primarily, cordycepin has been shown to directly enhance insulin secretion from pancreatic β-cells. Insulin, the sole hormone responsible for reducing blood glucose levels, is integral to maintaining glucose homeostasis in the body and is currently the primary treatment for diabetes (Stuhlmann et al., 2018). Sun et al. discovered that cordycepin, when used to treat both normal and oxidatively damaged INS-1 cells (a rat insulinoma cell line), could induce cell membrane depolarisation by promoting ATP production. This, in turn, amplified Ca2+ influx and ultimately improved insulin secretion in INS-1 cells (Sun et al., 2022). Furthermore, cordycepin has been shown to upregulate the genetic expression of pancreatic duodenal homeobox factor-1 (PDX-1) via activation of the Phosphatidylinositol-3 kinase/serine-threonine kinase (PI3K/Akt) pathway (Zhang et al., 2020; Sun et al., 2022). Liao and colleagues also confirmed through molecular docking studies that cordycepin can interact with the residues ASP836, ASP841, GLU880, and VAL882 in the active pocket of PI3K via hydrogen bond interactions, with a binding energy of −6.69 (Liao et al., 2020). PDX-1 is a critical transcription factor integral to insulin regulation. It plays a pivotal role in enhancing insulin expression and augmenting insulin secretion (Kaneto et al., 2005).

**FIGURE 7:**
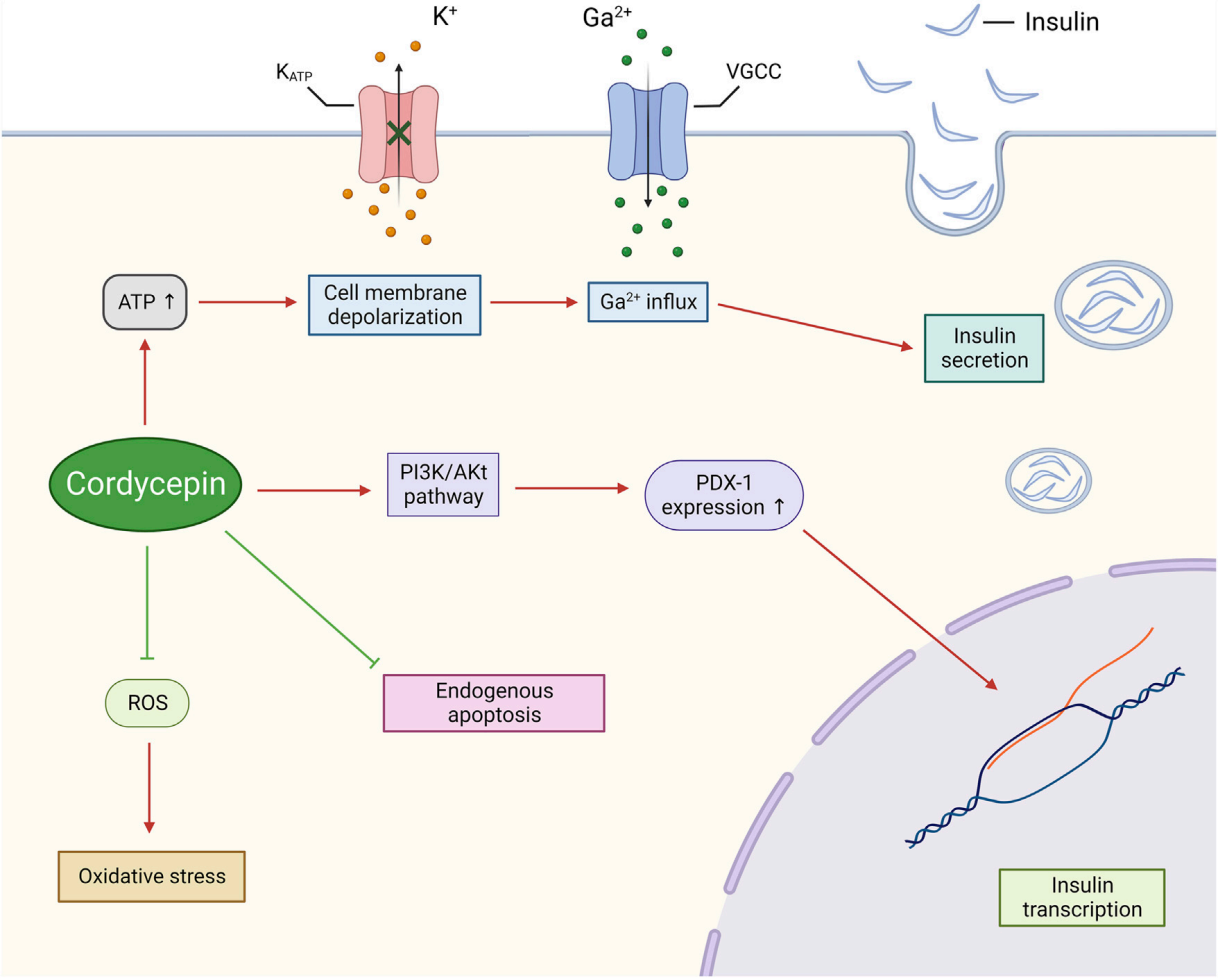
Cordycepin possesses the potential to safeguard pancreatic β-cells, whilst simultaneously enhancing their synthesis and secretion of insulin.. (Note: K_ATP_: ATP-sensitive Potassium Channel. VGCC: Voltage-gated Calcium Channel.)

It is widely recognised that prolonged exposure to free fatty acids or high glucose environments results in apoptosis of pancreatic islet cells, and the progression of diabetes is often marked by a progressive decrease in the number of β-cells (Piro et al., 2002). However, Yan et al. found that cordycepin could mitigate intracellular oxidative stress induced by mitochondria by reducing the production of reactive oxygen species (ROS), and inhibit endogenous apoptosis of INS-3 cells induced by it by inhibiting the JNK signalling pathway. This, in turn, preserves the number and functionality of pancreatic β-cells in both glucotoxic and lipotoxic environments (Yan et al., 2023) (Figure 7).

Cordycepin has also been identified as a potential therapeutic agent for insulin resistance. Insulin resistance is characterised by a significant reduction in glucose uptake and utilisation by the liver, muscle, and adipose tissue, leading to a rigid metabolic state that lacks the flexibility to alternate between glucose and fatty acid use (Turcotte et al., 2001; Liu et al., 2023). Cordycepin, during hepatic glucose metabolism, functions as an agonist of the AMPK pathway. This action inhibits the expression of key enzyme genes in the gluconeogenesis pathway, such as Phosphoenolpyruvate carboxykinase (PEPCK) and Glucose-6-phosphatase (G6Pase), by binding to their cAMP-responsive element (CRE), thereby suppressing hepatic glucose production (Lochhead et al., 2000; Zhou et al., 2001). Furthermore, Ma et al. demonstrated that cordycepin significantly enhances hepatic glycogen synthesis and promotes hepatic glucose utilisation (Ma et al., 2015). These combined effects contribute to the maintenance of glucose homeostasis. In skeletal muscle, chronic AMPK activation has been associated with the amelioration of muscle mitochondrial dysfunction in diabetic patients (Kelley et al., 2002), increased muscle glucose transporter protein 4 (GLUT4), hexokinase, and glycogen content, mimicking the effects of endurance training (Holmes et al., 1985), and overcoming metabolic inflexibility and insulin resistance. Moreover, Shin et al. discovered that cordycepin could inhibit the expression of genes promoting insulin resistance by deactivating NF-κB-dependent inflammatory responses in RAW 264.7 cells (a mouse macrophage cell line) stimulated by lipopolysaccharide (LPS), including 11β-hydroxysteroid dehydrogenase type 1 (11β-HSD1), peroxisome proliferators-activated receptor γ (PPARγ), and regulated upon activation normal T-cell expressed and secreted (RANTES) (Shin et al., 2009).

In a noteworthy study, Liu et al. revealed that cordycepin could also mitigate diabetes symptoms by fostering the growth of beneficial bacteria (e.g., increasing the Firmicutes/Bacteroidetes ratio), modulating gut microbiota structure, and enhancing metabolites and metabolic pathways associated with diabetic alterations (Liu et al., 2023). This discovery broadens the potential applications of cordycepin in diabetes management.

Traditional hypoglycemic agents like sulfonylureas, insulin, and dipeptidyl peptidase-4 (DPP-4) inhibitors focus on increasing insulin availability either by stimulating pancreatic secretion or by prolonging its action. However, these therapies often fail to address the underlying issues of oxidative stress and inflammation associated with diabetes, which cordycepin targets effectively. As mentioned above, studies suggest that cordycepin’s antioxidant properties may reduce oxidative stress and improve mitochondrial function in diabetic patients, offering a complementary route to standard treatments. This indicates a potential for combination therapy where cordycepin could be used alongside conventional drugs to enhance overall therapeutic outcomes and reduce side effects such as hypoglycaemia and weight gain commonly seen with insulin and sulfonylureas.

Further research into these synergistic effects could pave the way for novel integrative treatment protocols, positioning cordycepin as a valuable component of a multifaceted approach to diabetes management. The exploration of these possibilities is essential for developing more effective and personalized diabetes care strategies.

## 8 Summary and future perspectives

Historically, research has primarily focused on the direct anticancer properties of cordycepin. In this review, we delved deeply and systematically into the various pathways through which cordycepin exerts its anticancer effects. These pathways include not only direct mechanisms such as inhibition of biosynthesis, induction of apoptosis or autophagy, regulation of the cell cycle, and suppression of tumour invasion and metastasis, but also newly discovered indirect routes involving immune modulation of the tumour microenvironment. Furthermore, we have expanded the scope of this review to explore the potential impact of cordycepin on metabolic diseases, carefully examining the possible mechanisms by which cordycepin regulates hyperlipidaemia and diabetes, including its role in insulin sensitivity, glucose metabolism, and lipid regulation. This expansion broadens the potential application areas of this compound (Table 1).

**TABLE 1:** Application of cordycepin on tumours and metabolic diseases.

Diseases	Molecular mechanism	Target factor/Cell	Model	Results	References
Tumour	Suppression of RNA Synthesis and Termination of Protein Translation	RNA polymerases I, II, and III	L5178Y lymphoblasts and Ehrlich ascites-tumour cells	Termination of RNA synthesis	Klenow (1963), Hawley et al. (2020)
		Polyadenosine phosphorylation	NIH3T3 fibroblasts	Truncating the poly(A) tails of certain mRNAs, and obstructing the processing of mRNA at the 3’ end	Wong et al. (2010)
		AMPK	H1975 and PC9 non-small cell lung cancer cells	mTORC1↓, inhibition of protein translation	Wei et al. (2019)
	Induction of Neoplastic Cell Apoptosis and Augmentation of Cellular Autophagy	Bax	MDA-MB-231 and MCF-7 human breast cancer cells, NB-4 and U937 leukemia cells	Caspase-9↑, intrinsic apoptosis↑	Choi et al. (2011), Liao et al. (2015)
		MAPK	U87MG glioblastoma cells and MA-10 Leydig tumour cells	ERKs/JNKs/p38 MAPKs↑, intrinsic apoptosis↑	Pan et al. (2015), Baik et al. (2016)
		Death receptor	HT-29 colonic cancer cells and LNCaP prostate cancer cells	Forming the DISC, extrinsic apoptosis↑	Lee et al. (2013), Lee et al. (2014)
		β-cyclin signalling pathway	SKOV-3 and OVCAR-3 ovarian cancer cells	DKK↑, autophagy↑	Jang et al. (2019)
		ENT	A2780, SKOV-3, OVCAR-3, and TOV112D ovarian cancer cells	AMPK↑, autophagy↑	Yoon et al. (2022)
	Induction of Neoplastic Cell Cycle Arrest	Cyclin A/Cyclin E/CDK 2	NB-4 and U937 leukemia cells, BxPC-3 and AsPC-3 pancreatic cancer cells, NOZ and GBC-SD gallbladder cancer cells	Tumour cells are arrested in the S phase	Wang et al. (2014), Liao et al. (2015), Li et al. (2020)
		P21WAF1	HCT116 colon cancer cells, 5,637 and T-24 bladder cancer cells	Cyclin B/CDK1↓, tumour cells are arrested in the G2/M phase	Lee et al. (2009), Lee et al. (2010)
		p-ERK	ECA109 and TE-1 oesophageal cancer cells	Cyclin B/CDK1↓, tumour cells are arrested in the G2/M phase	Xu et al. (2019a)
		CHK1	HeLa cells	Cyclin B/CDK1↓, tumour cells are arrested in the G2/M phase	Seong da et al. (2016)
	Inhibition of Neoplastic Cell Invasion and Metastasis	Integrin α1 and FAK	U87MG and LN229 glioblastoma cells	Focal adhesion complexes↓	Hueng et al. (2017)
		IκBα	HepG2 and Huh7 liver cancer cells	CXCR4↓	(176)
		TWIST1/SNAI1/SNAI2/ZEB1	4T1 breast cancer cells	EMT↓	Wei et al. (2022)
		MMP-9	A549 and NCI-H460 lung cancer cells, HSC-4 oral squamous carcinoma cells	Degradation of the extracellular matrix↓	(Binlateh et al. (2022), 177)
		TJs, MMPs and TIMP-1/TIMP-2	LNCaP prostate cancer cells	Degradation of the extracellular matrix↓	Jeong et al. (2012)
	Modulation of the Tumour Microenvironment	NK cells	NK-92 cells and KKU-213A cells	Cytotoxicity against biliary cancer cells ↑	Panwong et al. (2021)
		T-cells	FM3A tumour-bearing C3H/He mice	Tregs formation ↓	Jeong et al. (2013)
		Dendritic cells	MC38/CT26 mouse tumour models	DCs counts ↑, DCs' antigen-presenting capability ↑	Chen et al. (2024)
		Colorectal cancer cells	MC38/CT26 mouse tumour models	PD-L1 expression ↓	Wu et al. (2023)
		Macrophages	MC38/CT26 mouse tumour models	(Combined with anti-CD47 antibody) Reactivation of macrophages, reversal of macrophage polarization	Feng et al. (2023)
		CD8+ T-cells	MC38/CT26 mouse tumour models	(Combined with CTLA-4 blockade) Enhanced efficacy and reduced exhaustion of CD8+ T-cells	Chen et al. (2023)
		CD8+ TILs and NK cells	MC38/CT26 mouse tumour models	(Combined with TIGIT blockade) NK cell activation ↑, CD8+ TIL activity ↑, Expression of exhaustion marker genes in NK cells and CD8+ TILs ↓	Chen et al. (2024)
Hyperlipidaemia	Through the AMPK Pathway	ACC1	Rat hepatocytes	Fatty acid synthesis↓	Viollet et al. (2009)
		ACC2	Rat hepatocytes	Fatty acid oxidation↑	Assifi et al. (2005)
		HMG-CoA reductase	Rat hepatocytes	Cholesterol synthesis↓	Viollet et al. (2009)
		MCD	Rat hepatocytes	Acetoacetyl-CoA oxidation↑	Assifi et al. (2005)
		mtGPAT	Rat hepatocytes	Triglyceride synthesis↓	Muoio et al. (1999)
		SREBP-1c/ChREBP	Mouse hepatocytes	Fatty acid synthase gene expression↓	Kawaguchi et al. (2002), Foretz et al. (2005)
	Reducing lipid droplets in adipocytes	White adipocytes	Rat adipocytes	Facilitating the transformation of white adipocytes into beige and brown adipocytes	Xu et al. (2019b)
		Fsp27, Rab5 and Rab11	Rat adipocytes	Inhibition of lipid droplet formation	Xu et al. (2019b)
		Perilipin1	Rat adipocytes	Stimulation of lipolysis	Xu et al. (2019b)
	Increasing LPL Activity	PRL	Rat GH-3 cells	Reduction of lipoprotein lipase activity via functional prolactin receptors	Li et al. (2018)
Diabetes	Promoting Insulin Secretion	ATP production	INS-1 insulinoma cells	Ca2+ influx↑, insulin secretion↑	Sun et al. (2022)
		PI3K/Akt pathway	INS-1 insulinoma cells	PDX-1↑, insulin expression↑	Zhang et al. (2020), Sun et al. (2022)
		ROS	INS-1 insulinoma cells	Mitigating mitochondrial-induced intracellular oxidative stress to protect pancreatic β-cells	Yan et al. (2023)
	Reducing Insulin Resistance	AMPK	HL1C rat hepatoma cells	PEPCK↓, G6Pase↓	Lochhead et al. (2000), Zhou et al. (2001)
		Hepatic glycogen	Alloxan-induced diabetic mice	Promoting hepatic glycogen synthesis	Ma et al. (2015)
		AMPK	Rat muscle cells	GLUT-4↑, Hexokinase↑	Holmes et al. (1985)
		NF-κB	RAW 264.7 macrophage cells	11β-HSD1↓, PPARγ↓, RANTES↓	Shin et al. (2009)
	Modulating the Gut Microbiota	Gut Microbiota	Type 2 diabetes mellitus mice	Firmicutes/Bacteroidetes ratio↑	Liu et al. (2023)

It must be acknowledged that the existing research on cordycepin is still not sufficiently comprehensive. For instance, most studies on cordycepin are conducted *in vitro*, and the transition from these studies to clinical applications remains slow. The pharmacological potential of cordycepin has not yet been fully realized in clinical settings, possibly due to challenges such as its short half-life and rapid metabolism. Additionally, the lack of large-scale human trials limits our understanding of its therapeutic range across different populations.

Looking ahead, there is an increasing interest in utilizing state-of-the-art research tools, including single-cell analysis, advanced fluorescent markers, and sophisticated imaging techniques (Miller and Weissleder, 2017; Jing et al., 2018; Rosenberg et al., 2018). These methodologies are expected to uncover the detailed mechanisms behind cordycepin’s therapeutic influence on both cancer and metabolic anomalies. By strategically altering cordycepin’s molecular structure, we aim to surpass its pharmacokinetic limitations, enhancing its efficacy and broadening its therapeutic scope. This will enable more comprehensive *in vivo* and clinical studies on cordycepin, evaluating its toxicity, safety, and potential clinical advantages. Such research is essential for the development of innovative treatments for tumours and metabolic disorders.
